# A Scalable Distributed Control Algorithm for Bearing-Only Passive UAV Formation Maintenance

**DOI:** 10.3390/s23083849

**Published:** 2023-04-10

**Authors:** Yuchong Gao, Huiqi Feng, Jiexiang Chen, Junhui Li, Zhiqing Wei

**Affiliations:** 1 School of Information and Communication Engineering, Beijing University of Posts and Telecommunication, Beijing 100876, China; 2 State Key Laboratory of Informational Photonics and Optical Communications, Beijing University of Posts and Telecommunications, Beijing 100876, China

**Keywords:** UAV formation, distributed control, formation maintenance, electromagnetic silence, bearing-only

## Abstract

Unmanned Aerial Vehicles (UAVs) can cooperate through formations to perform tasks. Wireless communication allows UAVs to exchange information, but for the situations requiring high security, electromagnetic silence is needed to avoid potential threats. The passive UAV formation maintenance strategies can fulfill the requirement of electromagnetic silence at the cost of heavy real-time computing and precise locations of UAVs. To pursue high real-time performance without the localization of UAVs, this paper proposes a scalable distributed control algorithm for bearing-only passive UAV formation maintenance. By minimizing necessary communication, pure angle information is applied to maintain UAV formations through distributed control, without the knowledge of the UAVs’ precise locations. The convergency of the proposed algorithm is proven strictly and the converging radius is derived. Through simulation, the proposed algorithm is proven to be suitable for a general case and demonstrates fast convergence speed, strong anti-interference capability, and high scalability.

## 1. Introduction

UAV formation is the coordinated flight of multiple UAVs used for tasks such as military operations, natural disaster management, and forestry protection [1,2]. There are two categories of current UAV formation control: motor coordination and coverage coordination [3]. Formation maintenance, which ensures the stability of UAV formation, is the core problem of motor coordination [4]. Achieving electromagnetic silence is essential for anti-interference and the safety of UAV formations, especially when there is limited communication capacity for electronic countermeasures.

Previously, extensive research has been conducted on maintaining UAV formations, which can be divided into two categories based on the communication method [4]. The first category is the centralized control method, where a control center is responsible for updating location information and transmitting control information within the UAV formation system. In [5], Brandão et al. proposed a multi-layer control scheme based on a control center, which achieves high accuracy but requires a significant amount of information exchange and imposes a heavy computing power requirement on the control center. The second category is the distributed control method, where each UAV exchanges information only with its neighbors. The leader–follower scheme [6,7,8,9,10] selects leader UAVs, and the remaining followers update their locations by exchanging information with the nearest leader. However, this method has poor robustness. The artificial potential field scheme [11] uses a natural potential function based on the structural constraints of the desired formation, but it risks falling into a local rather than a global optimum. The virtual structure scheme [12] regards UAV formation as a virtual rigid body and maintains it by adjusting each UAV to maintain a relative distance from a fixed point. However, its reliability and availability are poor because of its requirement for high communication quality and strong computing power. The behavior-based scheme [13] maintains UAV formation by presenting several UAV behaviors and corresponding behavior coordination rules, which has good scalability but poor anti-interference capability due to limited behavior. In addition to the traditional algorithms mentioned above, the rapid development of deep learning has enabled it to be applied in many fields [14,15,16,17,18], including the field of UAVs. Vision-based UAV formation maintenance has improved the performance of these algorithms, but still faces the problem of huge communication requirements [19,20] in scenarios with electromagnetic silence.

Since passive localization methods emit little electromagnetic signal, developing passive UAV formation maintenance strategies is of great significance. A natural UAV formation maintenance strategy relies on localization. However, passive localization requires heavy real-time computing and precise location information of at least one UAV. To save computing resources and pursue high real-time performance without UAV location information, a scalable distributed control algorithm for bearing-only passive UAV formation maintenance is proposed. The proposed algorithm adjusts UAVs to satisfy the geometry requirements using pure angle information. The simulation demonstrates fast convergence speed, strong anti-interference capability, and high scalability, along with the ability to be generalized to various formations. Furthermore, the convergence of the proposed algorithm is proved for circular formations, together with the derived convergence radius. The method works for a wide range of disturbance situations. It is worth noting that when the initial UAV radii are log-normally distributed [21], the convergence radius can converge to the initial preset radius as the UAV number *k* increases. The expectation of the difference between the convergence radius and the preset radius and the variance of the convergence radius are $o\left(\frac{1}{k}\right)$, which is equivalent to infinitesimals of $\frac{1}{k}$. This suggests strong anti-interference capability even when *k* is large.

## 2. Uav Formation Maintenance Methods

### 2.1. System Model

Maintaining a UAV formation involves a three-dimensional task that is simplified into a two-dimensional task on the horizontal plane. This is achieved by utilizing various physical actuators that monitor the speed and altitude, as noted in [22,23]. To simplify the task, a new coordinate system is constructed with the central UAV as the reference. The UAV formation is regarded as a whole, and only relative motion is considered, ignoring dynamic parameters such as UAV speed.

**Figure 1:**
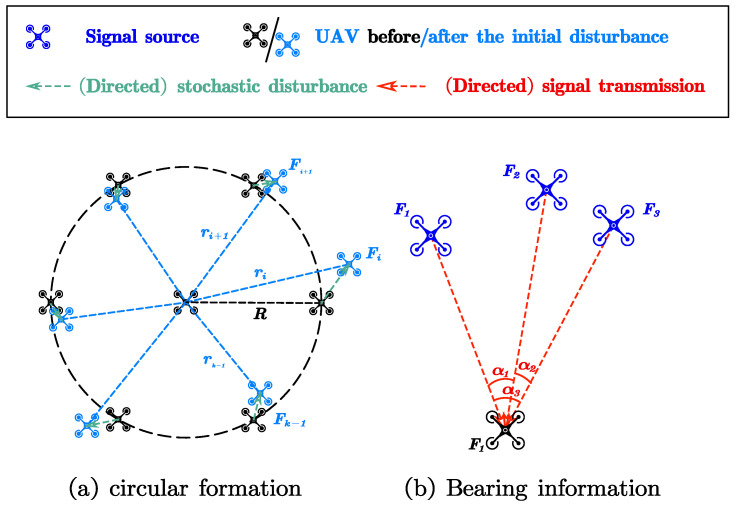
System model.

In Section 2.2, we adopt a circular formation. Note that the circular formation can be extended to a general formation in Section 2.3. Figure 1a depicts the preset circular UAV formation consisting of k+1,k≥2 UAVs, with $F_{0}$ located at the center of the circle. UAVs $F_{i},i=1,2,\cdots ,k$ are equally spaced on the circle with radius *R*. For brevity, we set $F_{k}=F_{1}$. The UAVs maintain the same altitude based on their altitude sensors, which consist of a Proportion Integration Differentiation (PID) controller and latitude sensor [22]. Initially, the UAVs on the circle are horizontally disturbed with radius $r_{i}$. We assume, following [21], that $r_{i}$ is log-normally distributed, i.e., $\ln r_{i}∼N\left(\ln R,\sigma^{2}\right)$, where σ represents the standard deviation.

To achieve electromagnetic silence, we adjust the UAVs’ locations using a bearing-only method. This means that only a few (at most three in the proposed algorithm) active UAVs transmit a signal at any given time, and the specified silent UAVs adjust their locations accordingly. We select only the angle information between UAVs as parameters for the formation. These angles can be measured through signals without specific communication content and ensure the safety of UAVs in electromagnetic silent scenarios. Each UAV maintains its own location relationship with other UAVs. Figure 1b illustrates that we stipulate the direction information as the angle between the passive UAV and any two active UAVs. For example, with UAVs $F_{1}$, $F_{2}$, and $F_{3}$ transmitting signals, the direction information received by $F_{4}$ is $\alpha_{1}$, $\alpha_{2}$, and $\alpha_{3}$, respectively.

Each UAV is equipped with a built-in synchronous clock to determine when it transmits/receives. Transmitting signals only at a given time minimizes the potential risk of information leakage and hostile interference, allowing for electromagnetic silence. All the information used in the algorithm is the angle between the radiation sources and the signal direction.

### 2.2. Circular UAV Formation Maintenance Method

In this section, a scalable distributed control algorithm for bearing-only passive UAV formation maintenance is introduced under the circular formation, which aims to allow the disturbed UAVs on the circle to detect and adjust their formation, and, finally, to keep themselves within the preset circle with radius *R*.

Intuitively, adjustments in both radial and tangential directions are sufficient, due to the symmetry of the circle. The algorithm consists of the following two steps.

**Figure 2:**
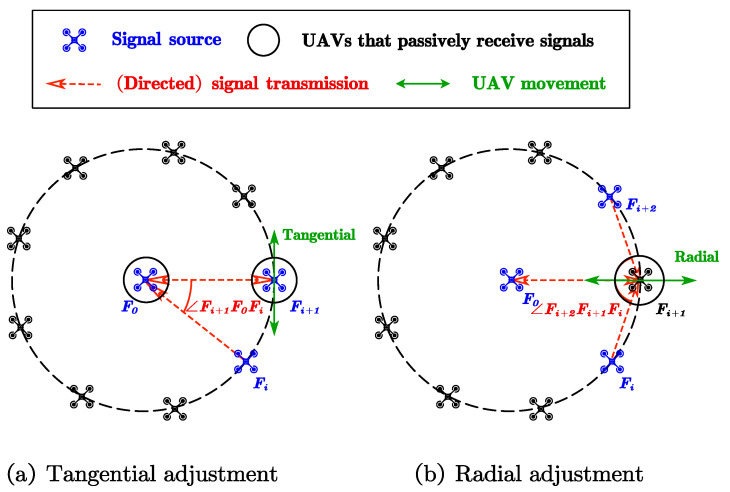
Circular formation algorithm.

**Figure 3:**
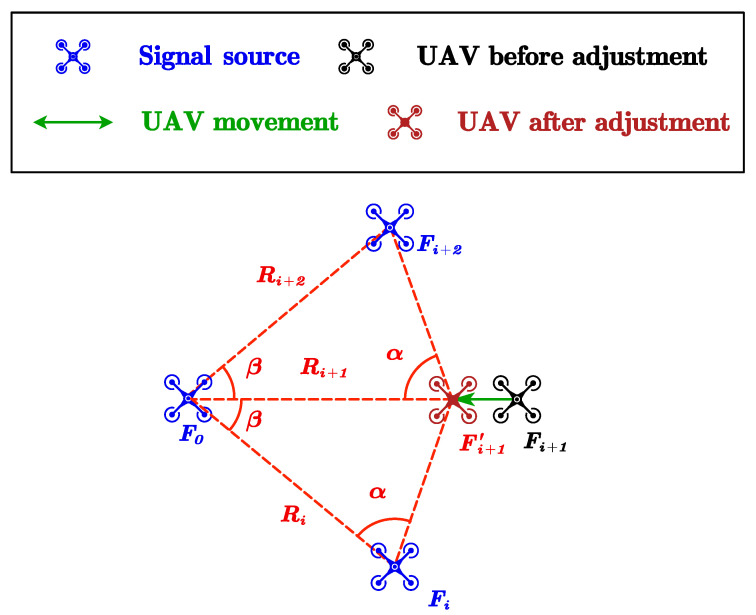
Geometry relation.

(1) Tangential adjustment
  In tangential adjustment, we choose two adjacent UAVs, $F_{i}$ and $F_{i+1}$, and the signal-emitting UAV $F_{0}$ in the center of the circle. $F_{0}$ and $F_{i+1}$ receive signals according to the clock. Next, $F_{i+1}$ finds the tangential direction as the direction perpendicular to the received signal. It moves in the tangential direction until the angle $∠F_{i}F_{0}F_{i+1}$ becomes $\frac{2\pi }{k}$. We repeat this adjustment until index *i* goes from 1 to *k*. After the tangential adjustment, adjacent UAVs have equal relative angles.
(2) Radial Adjustment
  In radial adjustment, we choose two UAVs $F_{i}$ and $F_{i+2}$ on the circle, $F_{0}$ in the center of the circle as the signal-emitting UAVs, and $F_{i+1}$ as the signal-receiving UAVs controlled by the clock. $F_{i+1}$ sets the radial direction as the direction of the received signals. It moves along the radial direction until the angle $∠F_{i}F_{i+1}F_{i+2}$ becomes $\frac{(k-2)\pi }{k}$. As shown in Figure 2, the triangle $▵F_{i+1}^{\prime }F_{0}F_{i+2}$ is similar to the triangle $▵F_{i}F_{0}F_{i+1}^{\prime }$, so the radius relationship of the UAVs satisfies $R_{i+1}=\sqrt{R_{i}R_{i+2}}$ in Figure 3. This adjustment repeats until index *i* goes from 1 to *k* for M rounds, which we discuss in Section 3. According to Theorem 1, the proposed algorithm converges, and the convergence radius after adjustment is $R=r_{n-1}^{\frac{1}{k}}\prod_{i=2}^{n-1}r_{i}^{\frac{1}{k}}$. The expectation and variance under initial log-normal distribution are $E(R)=Re^{\frac{k+2}{2k^{2}}\sigma^{2}}$ and $D(R)=\left(e^{\frac{k+2}{k^{2}}\sigma^{2}}-1\right)R^{2}e^{\frac{k+2}{k^{2}}\sigma^{2}}$, respectively. The difference between E(R) and preset radius and $D(R^{*})$ are $o\left(\frac{1}{k}\right)$, suggesting strong anti-interference capability, even for large values of *k*.

**Theorem** **1.** *Convergency and the convergence radius of the proposed circular UAV formation maintenance method.*

*k UAVs are equally spaced on the circle of radius R. Under initial disturbance, we set the logarithm of the radial distance of k UAVs relative to UAV $F_{0}$ as $a_{-1},a_{0},a_{1},\cdots ,a_{k-2}$ according to the order of UAV adjustment, and use $a_{i+k-2}\left(i\in N^{*}\right)$ to represent the logarithm of the radial distance of the UAVs relative to UAV $F_{0}$ after the ith adjustment. Through the recurrence relationship $a_{n}=\frac{a_{n-1}+a_{n-k+1}}{2}$, $a_{n}$ can converge to $\frac{a_{0}+a_{1}+\cdots +a_{k-3}+2a_{k-2}}{k}$ when n goes to infinity. If $a_{i}=\ln R+e_{i}∼N\left(\ln R,\sigma^{2}\right),i=-1,0,\cdots ,k-2$, we obtain the final convergent radius $\ln R^{*}=\frac{a_{0}+a_{1}+\cdots +2a_{k-2}}{k}∼N\left(\ln R,\frac{k+2}{k^{2}}\sigma^{2}\right)$ with its expectation $E\left(R^{*}\right)=Re^{\frac{k+2}{2k^{2}}\sigma^{2}}$ and the variance $D\left(R^{*}\right)=\left(e^{\frac{k+2}{k^{2}}\sigma^{2}}-1\right)R^{2}e^{\frac{k+2}{k^{2}}\sigma^{2}}$.*

**Proof.**  The proof is provided in Appendix A.  □

### 2.3. Extension to a General Formation

Inspired by the algorithm for circular formation, generalization is designed as follows, which demonstrates high scalability of the proposed algorithm.

We first summarize the optimizable formation requirements:

- There is an UAV in the center of the formation.
- The formation consists of *N* concentric circles of different radii.
- UAVs on the same circle are aimed to be equally spaced.

We divide formation into multiple concentric circles, and impose an angle restriction relationship based on independent adjustments of each circular formation according to the adjustment method introduced in Section 2.2. The angle restriction relationship should be specified according to the given formation. We take the conical formation as an example as follows.

**Figure 4:**
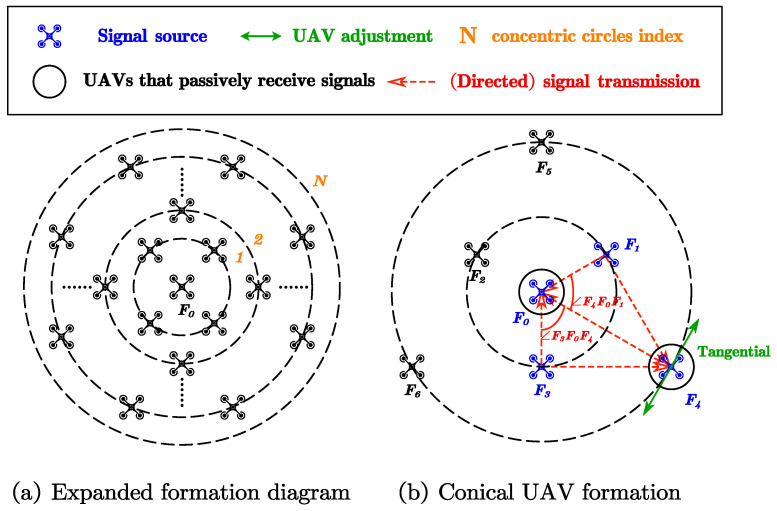
Expanded UAV formation.

As shown in Figure 4b, the conical UAV formation consists of $F_{0},F_{i},i=1,2,\cdots ,6$, where $F_{1},F_{2},F_{3}$ have a radius of $R_{1}$ and $F_{4},F_{5},F_{6}$ have a radius of $R_{2}=2R_{1}$. $F_{1},F_{2},F_{3}$ perform the tangential adjustment in Section 2.2. To ensure that the relative tangential location of the inner and outer circles remains unchanged, $F_{4}$ moves tangentially until $∠F_{1}F_{0}F_{4}=∠F_{3}F_{0}F_{4}$. $F_{4},F_{5},F_{6}$ then perform the tangential adjustment in Section 2.2. Finally, each UAV on the concentric circle performs the radial adjustment in Section 2.2. To prevent collisions, it is necessary to separate the UAVs on each concentric circle at different altitudes before the adjustment, where $F_{0}$ flies to the altitude of each concentric circle in turn, to participate in the adjustment of that circle.

## 3. Results

**Figure 5:**
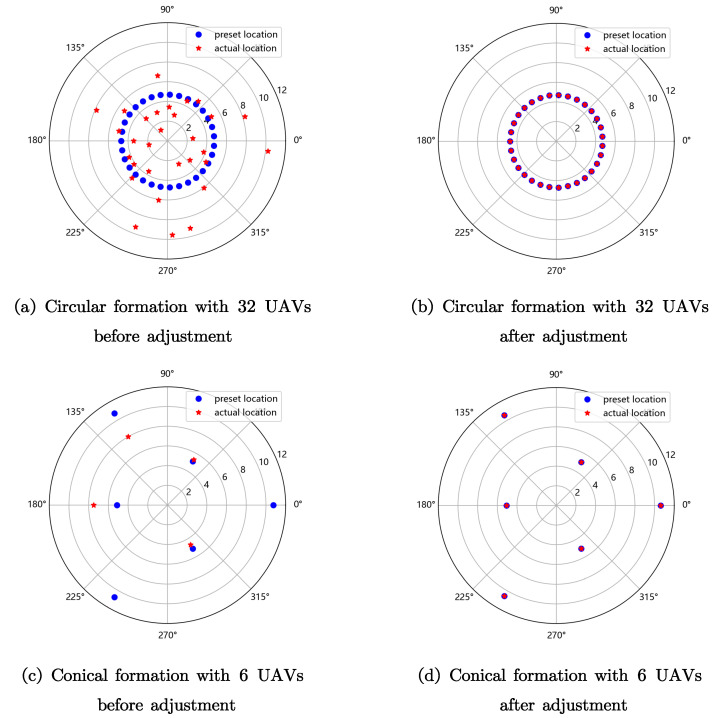
Schematic diagram for the algorithm.

In this section, we conduct simulations with Python to show the performance of the proposed algorithm, including the convergency of radius and converging speed, represented by precision, the number of necessary iterations, and summed moving distance. Figure 5 demonstrates the results of the proposed algorithm for circular and conical UAV formation maintenance, where the center UAV is not drawn for brevity.

We first define the radial error as $e_{R}=\frac{1}{k}\sum_{i=1}^{k}{\left(R_{i}-\bar{R}\right)}^{2}$, where $\bar{R}=\frac{1}{k}\sum_{i=1}^{k}R_{i}$. When the error $e_{R}$ is less than terminating error $E_{R}=\left\{10^{-3}\;m,10^{-5}\;m,10^{-7}\;m,10^{-9}\;m\right\}$, the adjustment is terminated. The parameters are set as follows Table 1. We set the UAV number *k*, ranging from 3 to 31, which is in line with actual situations, preventing the UAVs from being too sparse or too densely distributed. We set the initial radius as R=5 m, which is an empirically reasonable actual distance between UAVs. The disturbed initial radius *Y* of the UAVs follows the logarithmic normal distribution, where the mean value is ln5 m and the standard deviation is 0.5 m, i.e., $\ln Y∼N(\ln5\;m,0.5^{2}\;m)$. For each UAV number, we repeated the simulation 100 times. In the simulation figures, the curve joints the average values of the scatter diagram.

**Figure 6:**
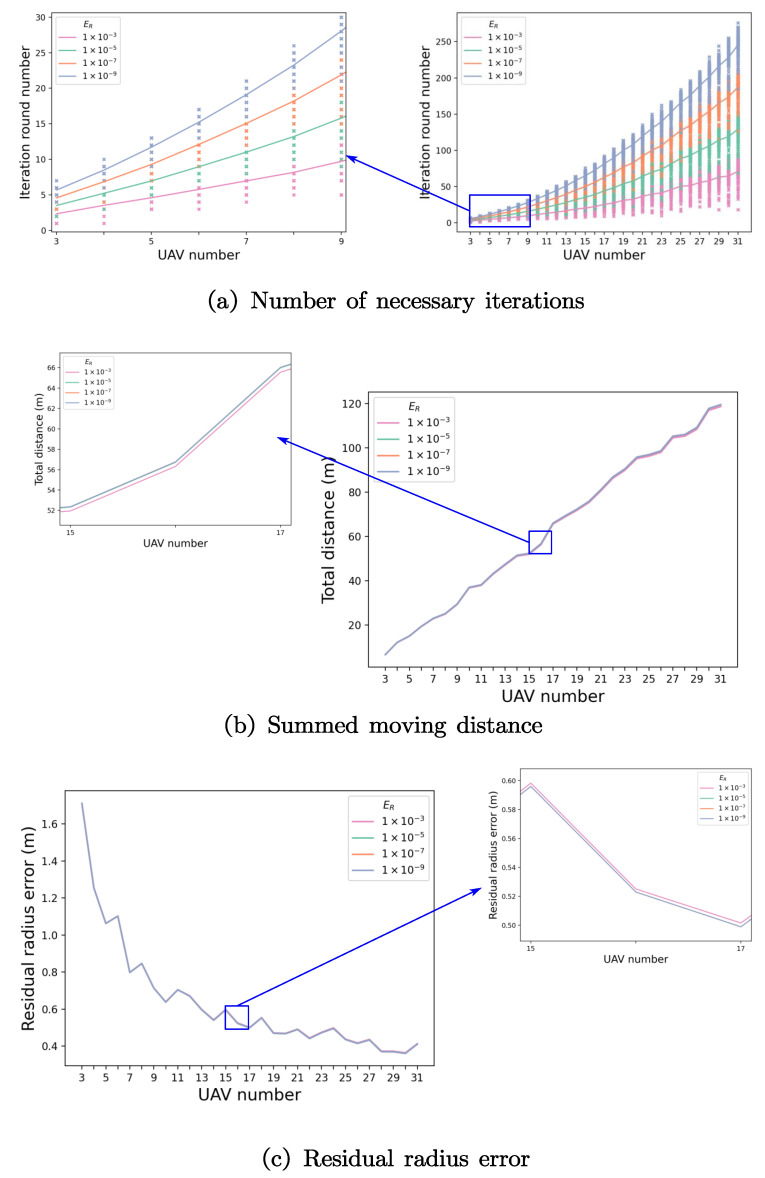
The performance of the algorithm.

As shown in Figure 6a, the iteration number required for convergence has a nonlinear relationship with the UAV number. When the UAV number is less than 10, it only takes less than 10 iterations to achieve a radial error of less than 0.001, which proves that our algorithm has a fast convergence speed when UAV number is small. Besides, the data fluctuate little for repeated simulations, suggesting that the iteration number for convergence does not change much with the initial disturbance, and our algorithm has strong anti-interference capability. The design criteria for iteration number *M* in Section 2.2 is given as follows: according to the disturbance and formation, the iteration number *M* can be selected according to different accuracy requirements through simulation.

As shown in Figure 6b, the summed moving distance of UAVs before convergence increases proportionally with the UAV number, suggesting that the average travel distance of each UAV remains constant with the increase in UAV number, which means that the performance does not degrade as the UAV number increases.

Figure 6c depicts the change in residual radius error, i.e., the root mean square error (RMSE) between each UAV’s convergence radius and initial preset radius, against UAV numbers. The final convergence radius approaches the initial radius as the number of UAVs increases, confirming the expectation of convergence radius $E\left(R^{*}\right)=Re^{\frac{k+2}{2k^{2}}\sigma^{2}}$ under log-normal distribution. This suggests that tangential adjustment and radial adjustment in Section II.B are sufficient for UAV formation maintenance when *k* is large.

Overall, our algorithm can converge quickly within a small number of iterations when the number of UAVs is small. However, the convergence radius of individual UAV may deviate slightly from the pre-set value. As the number of UAVs increases, the average flight distance of each UAV before convergence remains almost the same, and the converged radius becomes closer to the initial radius. This indicates that the proposed method has good potential for scalability with respect to UAV number.

It should be noted that we did not conduct comparative experiments in this section, as to the best of our knowledge, reference [23] is the only paper related to our work. The study aimed to achieve UAV formation maintenance in electromagnetic silence scenarios by minimizing the number of signal-emitting sources, and the assumptions made in that paper are different from ours. They imposed strong assumptions with limitations, where the UAVs obtain their initial position information, and the performance of their algorithm was only verified in one case. In comparison, our proposed algorithm is more suitable for general cases and has excellent performance.

## 4. Discussion

In this contribution, we focused on a general method and principle of UAV formation, without considering a specific UAV model [19,24]. For simplicity, we ignored the processing details and potential transmit error of radiation source signals in comparison to [23]. We also did not model the flight process of UAVs in detail, such as travel speed and flight direction, assuming immediate adjustment to avoid lengthy discussions. A detailed dynamic model including the above factors would be favorable in the future.

## 5. Conclusions

To pursue UAV formation maintenance in electromagnetic silence, we have proposed a bearing-only algorithm that has been verified through simulations, showing fast convergence speed, strong anti-interference capability, and high scalability. For circular formation, we have rigorously proven the convergence of the algorithm and derived the convergence radius $R^{*}=r_{n-1}^{\frac{1}{k}}\prod_{i=2}^{n-1}r_{i}^{\frac{1}{k}}$. The expectation and variance of the convergence radius under initial log-normal distribution are $E(R)=Re^{\frac{k+2}{2k^{2}}\sigma^{2}}$ and $D(R)=\left(e^{\frac{k+2}{k^{2}}\sigma^{2}}-1\right)R^{2}e^{\frac{k+2}{k^{2}}\sigma^{2}}$, respectively. The difference between E(R) and preset radius and D(R) is $o\left(\frac{1}{k}\right)$, suggesting a strong anti-interference capability even when *k* is large. Furthermore, we have extended the proposed algorithm to general formations, and the generalization has demonstrated high scalability. It is worth noting that electromagnetic silence improves safety by avoiding most signal transmission and greatly reducing the amount of signal radiation. A more focused study on the necessary transmission time and flux is worth further investigation. Additionally, we plan to apply the proposed algorithm to specific UAV types and conduct experimental verification.

## Figures and Tables

**Table 1:** Table of the experimental result with $E_{R}=10^{-3}$ m.

UAV Number	Iteration Round	Total Distance (m)	Residual Radius Error (m)
3	2.36±0.57	6.674±4.196	1.712±1.409
4	3.54±0.61	12.188±7.110	1.256±1.073
5	4.61±0.79	14.974±7.789	1.063±0.942
6	5.78±0.84	19.297±8.554	1.103±0.834
7	6.98±1.10	22.877±8.406	0.798±0.604
8	8.18±1.19	24.987±9.396	0.847±0.625
9	9.72±1.73	29.326±10.791	0.714±0.521
10	11.39±1.91	36.780±12.244	0.639±0.545
11	13.17±2.43	37.895±12.996	0.705±0.575
12	14.63±2.87	42.959±15.122	0.672±0.580
13	16.63±3.48	47.099±13.320	0.598±0.469
14	18.89±3.85	51.126±13.116	0.542±0.417
15	20.21±4.61	51.947±15.804	0.598±0.525
16	22.40±5.03	56.316±15.271	0.525±0.389
17	25.42±5.23	65.557±17.611	0.501±0.389
18	27.44±6.03	68.767±19.264	0.554±0.458
19	31.12±6.43	71.800±17.853	0.472±0.391
20	32.32±7.98	75.298±17.465	0.469±0.287
21	36.47±7.59	80.581±20.263	0.492±0.387
22	39.72±8.34	86.260±17.063	0.444±0.376
23	41.29±9.53	89.852±19.133	0.475±0.407
24	45.88±9.18	95.121±20.232	0.499±0.367
25	50.44±10.63	96.204±18.988	0.438±0.296
26	51.34±12.30	97.896±21.835	0.417±0.284
27	55.61±10.76	104.467±18.121	0.436±0.321
28	58.18±13.24	105.222±20.089	0.373±0.260
29	63.11±11.61	108.339±21.412	0.373±0.273
30	64.38±15.91	116.879±21.363	0.364±0.280
31	71.11±15.17	118.583±25.774	0.413±0.280

## Data Availability

Data available on request from the authors.

## Abbreviations

The following abbreviation is used in this manuscript:

UAV	Unmanned Aerial Vehicle
PID	Proportion Integration Differentiation
RMSE	Root Mean Square Error

## Appendix A. Proof of Theorem 1

(A1) $$\begin{array}{c} a_{n}=\frac{a_{n-1}+a_{n-k+1}}{2} \end{array}$$

is called constant-coefficient linear homogeneous recurrence equation [25]. The characteristic polynomial is

(A2) $$\begin{array}{c} C\left(x\right)=2x^{k-1}-x^{k-2}-1 \end{array}$$

and the characteristic equation is

(A3) $$\begin{array}{c} 2x^{k-1}-x^{k-2}-1=0 \end{array}$$

where the k−1 roots of the characteristic equation are $x_{1}=1,x_{2},x_{3},\cdots ,x_{k-1}$. There are k−2 real roots of

(A4) $$\begin{array}{c} C^{\prime }\left(x\right)=\left(2k-2\right)x^{k-2}-\left(k-2\right)x^{k-3} \end{array}$$

as

(A5) $$\begin{array}{c} x_{1}^{\prime }=x_{2}^{\prime }=\cdots =x_{k-3}^{\prime }=0,x_{k-2}^{\prime }=\frac{k-2}{2k-2} \end{array}$$

As for the characteristic equation C(x)=0, when *k* is odd, there are two real roots $x_{1}=1,x_{2}$, where $-1<x_{2}<0$. When *k* is even, there is one real root $x_{1}=1$. Obviously, $C^{\prime }\left(x\right)=0$ and C(x)=0 have no common root, that is, the k−1 roots of the characteristic equation are different from each other. We get the general equation as

(A6) $$\begin{array}{c} a_{n}=\sum_{i=1}^{k-1}A_{i}x_{i}^{n} \end{array}$$

which yields the following equations with substituting the initial value:

(A7) $$\begin{array}{cc} \; & a_{0}=A_{1}+A_{2}+\cdots +A_{k-1}\\ \; & a_{1}=A_{1}+A_{2}x_{2}+\cdots +A_{k-1}x_{k-1}\\ \; & a_{2}=A_{1}+A_{2}x_{2}^{2}+\cdots +A_{k-1}x_{k-1}^{2}\\ \; & \cdots \\ \; & a_{k-2}=A_{1}+A_{2}x_{2}^{k-2}+\cdots +A_{k-1}x_{k-1}^{k-2} \end{array}$$

If $|x_{i}|<1,i=2,3,\cdots ,k-1$, there must be

(A8) $$\begin{array}{c} \lim_{n->\infty }a_{n}=A_{1} \end{array}$$

so that we first prove that the absolute value of roots of characteristic equation except $x_{1}=1$ are less than 1. We substitute

(A9) $$\begin{array}{c} x_{0}=\alpha e^{i\theta },\alpha >0 \end{array}$$

into the characteristic equation

(A10) $$\begin{array}{c} 2x_{0}^{k-1}-x_{0}^{k-2}-1=0 \end{array}$$

to get

(A11) $$\begin{array}{c} 2\alpha^{k-1}e^{j\theta (k-1)}=\alpha^{k-2}e^{j\theta (k-2)}+1 \end{array}$$

We then take the modulus of the left and right sides of the equation to get

(A12) $$\begin{array}{c} 2\alpha^{k-1}=\left|\alpha^{k-2}e^{j\theta (k-2)}+1\right|\leq \alpha^{k-2}+1 \end{array}$$

If α>1, then $2\alpha^{k-2}<2\alpha^{k-1}\leq \alpha^{k-2}+1$, whose solution is α<1, which does not match the assumption. If α=1, there must be θ=2mπ,m∈N, which is the root $x_{1}$. Therefore, there must be α<1, that is, $|x_{i}|<1,i=2,3,\cdots ,k-1$. Then $A_{1}$ is solved according to Cramer’s law [26]

(A13) $$A_{1}=\frac{D_{1}}{D}=\frac{\left|\begin{array}{cccc} a_{0} & 1 & \cdots & 1\\ a_{1} & x_{2} & \cdots & x_{k-1}\\ \vdots & \vdots & \ddots & \vdots \\ a_{k-2} & x_{2}^{k-2} & \cdots & x_{k-1}^{k-2} \end{array}\right|}{\left|\begin{array}{cccc} 1 & 1 & \cdots & 1\\ 1 & x_{2} & \cdots & x_{k-1}\\ \vdots & \vdots & \ddots & \vdots \\ 1 & x_{2}^{k-2} & \cdots & x_{k-1}^{k-2} \end{array}\right|}$$

According to Veda’s theorem, we get the following formulas

(A14) $$\begin{array}{c} \sum_{2\leq i_{1}<i_{2}<...<i_{p}\leq k-1}\prod_{j=1}^{p}x_{i_{j}}={\left(-1\right)}^{p}\frac{1}{2},\cdots ,p=1,2,\cdots ,k-2. \end{array}$$

The denominator of $A_{1}$ is

(A15) $$\begin{array}{cc} D & =\left|\begin{array}{cccc} 1 & 1 & \cdots & 1\\ 1 & x_{2} & \cdots & x_{k-1}\\ \vdots & \vdots & \ddots & \vdots \\ 1 & x_{2}^{k-2} & \cdots & x_{k-1}^{k-2} \end{array}\right|\\ \; & =\prod_{2\leq j<i\leq k-1}\left(x_{i}-x_{j}\right)\prod_{2\leq i\leq k-1}\left(x_{i}-1\right)\\ \; & =\prod_{2\leq j<i\leq k-1}\left(x_{i}-x_{j}\right)\sum_{p=0}^{k-2}\left[{\left(-1\right)}^{k-2-p}\sum_{2\leq i_{1}<i_{2}<...<i_{p}\leq k-1}\prod_{j=1}^{p}x_{i_{j}}\right]\\ \; & =\prod_{2\leq j<i\leq k-1}\left(x_{i}-x_{j}\right)*\sum_{p=0}^{k-2}\left[{\left(-1\right)}^{k-2-p}*\left\{\begin{array}{c} {\left(-1\right)}^{p}*\frac{1}{2}\left(p\neq 0\right)\\ 1(p=0) \end{array}\right.\right]\\ \; & ={\left(-1\right)}^{k-2}\prod_{2\leq j<i\leq k-1}\left(x_{i}-x_{j}\right)\frac{k}{2} \end{array}$$

We expand the numerator of $A_{1}$ according to the first column and take the product of the k − 1 determinants of the molecule and its coefficient ${\left(-1\right)}^{i},\left(0\leq i\leq k-2\right)$ as $V_{0},V_{1},\cdots ,V_{k-2}$, where

(A16) $$\begin{array}{cc} V_{k-2-p} & ={\left(-1\right)}^{k-2-p}\prod_{2\leq j<i\leq k-1}\left(x_{i}-x_{j}\right)\sum_{2\leq i_{1}<i_{2}<...<i_{p}\leq k-1}\prod_{j=1}^{p}x_{i_{j}}\\ \; & ={\left(-1\right)}^{k-2-p}\prod_{2\leq j<i\leq k-1}\left(x_{i}-x_{j}\right)*\left\{\begin{array}{c} {\left(-1\right)}^{p}*\frac{1}{2}\left(p\neq 0\right)\\ 1(p=0) \end{array}\right.\\ \; & ={\left(-1\right)}^{k-2}\prod_{2\leq j<i\leq k-1}\left(x_{i}-x_{j}\right)\times \left\{\begin{array}{c} \frac{1}{2}\left(p\neq 0\right)\\ 1(p=0) \end{array}\right. \end{array}$$

(A17) $$\begin{array}{c} D_{1}={\left(-1\right)}^{k-2}\prod_{2\leq j<i\leq k-1}\left(x_{i}-x_{j}\right)*\left(\frac{a_{0}}{2}+\frac{a_{1}}{2}+\cdots +\frac{a_{k-3}}{2}+a_{k-2}\right) \end{array}$$

We get the constant term as

(A18) $$\begin{array}{c} A_{1}=\frac{a_{0}+a_{1}+\cdots +a_{k-3}+2a_{k-2}}{k} \end{array}$$

Since the absolute values of the remaining roots are all less than 1, $a_{n}$ can converge when *n* tends to infinity, so that

(A19) $$\begin{array}{c} \lim_{n->\infty }a_{n}=A_{1}=\frac{a_{0}+a_{1}+\cdots +a_{k-3}+2a_{k-2}}{k} \end{array}$$

Given the probability density function of the log-normal distribution, the expectation and variance of the radius of convergence are derived as $Re^{\frac{k+2}{2k^{2}}\sigma^{2}}$ and $\left(e^{\frac{k+2}{k^{2}}\sigma^{2}}-1\right)R^{2}e^{\frac{k+2}{k^{2}}\sigma^{2}}$, respectively.
